# Mutations in the Receptor Binding Domain of Severe Acute Respiratory Coronavirus-2 Omicron Variant Spike Protein Significantly Stabilizes Its Conformation

**DOI:** 10.3390/v16060912

**Published:** 2024-06-04

**Authors:** Michael H. Peters

**Affiliations:** Department of Chemical and Life Science Engineering, Virginia Commonwealth University, 601 West Main Street, Richmond, VA 23284, USA; mpeters@vcu.edu; Tel.: +1-804-828-7790

**Keywords:** SARS-CoV-2, coronavirus, spike protein, Omicron, receptor binding domain, conformational stability, biomechanical stability, protein stability, tumbling corrections

## Abstract

The Omicron variant and its sub-lineages are the only current circulating SARS-CoV-2 viruses worldwide. In this study, the conformational stability of the isolated Receptor Binding Domain (RBD) of Omicron’s spike protein is examined in detail. The parent Omicron lineage has over ten mutations in the ACE2 binding region of the RBD that are specifically associated with its β hairpin loop domain. It is demonstrated through biophysical molecular computations that the mutations in the β hairpin loop domain significantly increase the intra-protein interaction energies of intra-loop and loop–RBD interactions. The interaction energy increases include the formation of new hydrogen bonds in the β hairpin loop domain that help stabilize this critical ACE2 binding region. Our results also agree with recent experiments on the stability of Omicron’s core β barrel domain, outside of its loop domain, and help demonstrate the overall conformational stability of the Omicron RBD. It is further shown here through dynamic simulations that the unbound state of the Omicron RBD remains closely aligned with the bound state configuration, which was not observed for the wild-type RBD. Overall, these studies demonstrate the significantly increased conformational stability of Omicron over its wild-type configuration and raise a number of questions on whether conformational stability could be a positive selection feature of SARS-CoV-2 viral mutational changes.

## 1. Introduction

**Figure 1:**
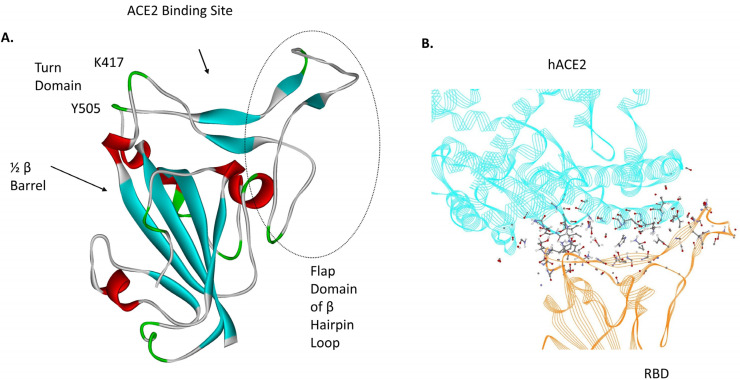
(**A**) The RBD of unbound WT SARS-CoV-2 (PDB ID: 6Z97; secondary structure after 0.3 μs simulation) showing the different structural regions and the β hairpin loop domain. (**B**) hACE2 bound to WT RBD (PDB ID: 6M17; 0.3 μs simulation); ball and stick atom representations are the dominant atom–atom interaction energies among its residues [1]. See supplementary information in Ref. [1] for quantitative details.

In a recent study, the dynamic features of the receptor binding domain (RB) of the Wuhan, wild-type (WT) SARS-CoV-2 spike protein (genus *betacoronavirus*; subgenus *sarbecovirus*) were examined using ab initio, all-atom molecular dynamics [1] in both its bound state with human Angiotensin Converting Enzyme 2 (hACE2) and unbound state. The RBD, which is part of the ectodomain of the trimeric spike protein, has a flap structure that may play a critical role in the binding dynamics of the virus to hACE2. The flap (loop) itself is part of a “beta hairpin loop” protein structural motif that overall contains turns and beta strands that *are* directly associated with hACE2 binding residues of the RBD (Figure 1). The flap of the RBD in its spike protein “Up” state was shown to oscillate between a binding site obstruction orientation (closed) and an unobstructed state (open) at an approximate time period of 100 ns. This β hairpin loop subdomain of the RBD consists of two centered, anti-parallel beta strands (L492-S494, L452-R454) flanked on one side by a highly flexible loop (L455-P491). The opposite flank with turns near residues T500-N501 and N439-N422 is stabilized by the distal beta strands, residues N394-I402 and Y508-E516. These distal beta strands are part of a half β barrel motif (five strands in total) that creates a stable RBD structure in isolation from its parent spike protein chain, as shown in Figure 1.

Currently, sub-lineages of the Omicron variant (B.1.1.529), which first appeared in late 2021, are the only circulating variants of SARS-CoV-2 with over 600 sub-lineages that have been identified in circulation [2]. The parent Omicron variants (sub-lineages BA.1 and BA.2) [3] contain an impressive 10 mutations in their roughly 60-residue ACE2 binding region as listed in Table 1. The corresponding binding partner, ACE2 residues are primarily from the α-1 helix of ACE2, as also shown in Table 1, and represent a typical helical binding pattern as a result of its three to four residues per turn configuration. A total of 36 residues from the WT RBD binding region were previously identified as associated with dominant atom–atom energetic interactions with ACE2 residues [1]. Interestingly, all 10 Omicron mutation residues are present in this list and none outside of it, potentially demonstrating positive selection for ACE2 binding of these specific mutations. For example, it has been shown that the mutation N501Y increases its interaction with ACE2 by forming a new, more favorable ACE2 partner residue Y41 [4,5] with a shift from G354, and there are multiple independent experimental and molecular dynamic studies demonstrating that Omicron mutations of the RBD increase its binding strength to hACE2 [6,7,8,9,10,11,12] over WT.

Recently, using Atomic Force Microscopy (AFM) and mutational screening, the mutation S373P was experimentally shown to be primarily responsible for an observed overall significant increase in the biomechanical stability of the isolated Omicron RBD [13]. It was demonstrated that the S373P mutation significantly increases the stability of the ½ β-barrel structural motif through the creation of new hydrogen bonds between its β strands in this domain.

Because of the potential importance of the β hairpin loop domain, this study examines what role mutations in the spike protein associated with the SARS-CoV-2 Omicron variant may play in the conformational dynamics of the β hairpin loop structure and across the RBD in general. The dynamics of the conformational states of the RBD, Omicron, and WT bound to hACE2 are also examined noting differences between the free state and bound state of the RBD. It will be demonstrated that Omicron’s RBD mutations result in significantly improved biomolecular mechanical stability and a more bound-state-like conformation, which *could* be a factor in its circulation persistence in addition to its stronger binding to hACE2 and other possible transmissibility factors [14].

The Omicron variant and its sub-lineages are currently the dominant circulating variants in SARS-CoV-2 infections worldwide and are the single focus of this study. Studies of other SARS-CoV-2 variants such as α, β, γ, Δ, etc. are beyond the scope of this study.

## 2. Materials and Methods

### 2.1. Molecular Dynamics

Explicit solvent molecular dynamics (MD) simulations were performed using the NAMD2 program [15] and CHARMM GUI interface [16]. The CHARMM36m force field was used along with TIP3P water molecules to explicitly solvate the proteins [16]. Simulations were performed maintaining the number of simulated particles, pressure, and temperature (the NPT ensemble) constant. The Langevin piston method was used to maintain a constant pressure of 1 atm. Periodic boundary conditions were employed for a water box simulation volume with the particle mesh Ewald (PME) method for water interactions and a 20 Å distance between the simulated protein edge and the water box edge. The integration time step was 2 femtoseconds with the total simulation time being approximately 0.3 microseconds (or 150 million total time steps) for each protein system analyzed. All our protein simulations were conducted under physiological conditions (37 °C, pH of 7.4, physiological ionic strength) [17]. Output *.pdb files were generated using VMD [18].

All simulations included the three cysteine disulfide bonds of the RBD (480–488, 336–361, and 379–432). Omicron RBD initial structure files were taken from PDB ID 7WK3 [19] and 7THK [20]; Wuhan WT structure files were taken from 6VSB [21] and 6Z97 [22]. The RBD segment was parsed from the “Up” chain of these particular structure files (all files exhibited a “One Up” chain configuration). Missing residues of 6VSB were added via the CHARMM GUI interface. RBD-hACE2 bound structures were taken from PDB IDs: 6M17 [23], 6M0J [24], 79TL [25], and 7U0N [26]. Note that the resolution of these structures is on the average of 2.9Å.

### 2.2. RMSF and Order Parameter

Root mean square fluctuation (RMSF) data were obtained for C-alpha atom positions over a period of 250 nanoseconds after discarding the first 50 ns. equilibration period of the 0.3 μs. total simulation time, which was previously determined as being greater than the time required to reach a dynamic structural equilibrium from the initial *.pdb structure file [17].

More precisely, the RMSF is determined from

$$RMSF=\sqrt{\frac{1}{N}\sum_{k=1}^{N}\left(∆x_{k}^{2}+∆y_{k}^{2}+∆z_{k}^{2}\right)}$$

where $∆x_{k}=x_{k}\;$−$\underline{\;x}$,$\;∆y_{k}=y_{k}\;$− $\underline{y}$, etc., with $x_{k}$ denoting the x-position of a specific C_α_ atom for each kth time and $\underline{x}$ is the average x position over the time ensemble, etc. (N is the total time ensemble number; 150 snapshots). As before [17], because of overall protein drift in the lab frame, all x positions for the spike protein are measured relative to the COM of the body frame, which is taken to be approximately residue D398 in the ½ β barrel domain (nearly rigid domain). 

Note that the RMSF values are indicative of internal translational fluctuations relative to the center of mass of the protein. In addition, atoms undergo angular position changes about the center of mass, such as with hinge motions in proteins. Rotational changes and fluctuations are established through so-called order parameters or rotational “stiffness” typically looking at the N-H bond vector of the backbone atoms of each residue.

Here, order parameters were calculated for N-H bond vector rotational correlations for each residue based on 250 total “snapshots”. Again, data for the first 50 ns. were discarded. More precisely, the order parameter, S^2^, is calculated from the plateau values of the rotational correlation function

$$C(\Delta t)={<P_{2}(cos\;\theta )>}_{∆t}$$

where $P_{2}$ is the second-order Legendre polynomial, $\cos\theta =n\left(t+∆t\right)\cdot n\left(t\right)$, and n(t) is a unit vector along the N-H bond vector at any time t; < > denotes an ensemble average for each time interval Δt. For rigid proteins, the order parameter would be one. For uniformly random orientations between π/2 and −π/2, the order parameter would be zero. Following our previous study, the plateau values occur at a Δt of approximately 100 ns. The algorithm developed for generating RMSF and order parameter data is given in Supplementary Information (S4). Because of the relatively small molecular weight RBD, approximate corrections to tumbling motions (macromolecular rotational diffusion) were invoked for the order parameter and rotational correlation analysis, as shown in the Supplementary Information (Supplement S4). 

### 2.3. All-Atom Energetic Mappings

Previously [27], we analyzed the non-covalent or non-bonded atom–atom interactions across two independently published structure files (PDB ID: 6VSB and 6VYB; one up and two down protomers) for the SARS-CoV-2 trimeric spike protein using the open source energy mapping algorithm developed by Krall et al. [28]. This spatial and energetic mapping algorithm efficiently parses the strongest or most dominant non-covalent atom–atom interactions (charge and partial atomic charge, Born, and van der Waals forces), according to empirically established parsing criteria, based on the ab initio AMBER03 force field model. 

More specifically, the non-covalent interaction potential energy between any two atoms (i and j) at a distance r_ij_ apart is expressed as

(1) $$\varphi_{ij}=\epsilon_{ij}\left[{\left(\frac{R_{ij}^{min}}{r_{ij}}\right)}^{12}-2{\left(\frac{R_{ij}^{min}}{r_{ij}}\right)}^{6}\right]+\frac{q_{i}q_{j}}{r_{ij}}$$

which represents Born repulsive, van der Waals attractive, and Coulombic interactions in order. The parameters ε_ij_, R_ij_^min^, and the charge or partial charge q_i_ or q_j_ depend on the specific atoms of the protein. Following our previous studies, the parsing criteria is taken as the upper limit of −0.1 kT units (k is Boltzmann’s constant and T is the absolute temperature) for Lennard-Jones (van der Waals) criteria and −0.3 kT units for Coulombic interactions, although lower values can also be specified in the analysis part of the mappings in order to further refine the results [28]. These limits help ensure that only the stronger non-covalent interactions below the thermal or kinetic energy of any atom (~kT) are identified. All results are reported here as φ_ij_/kT with more negative results indicative of stronger interaction attractive forces and greater conformational stability. Note that in this analysis the van der Waals interaction forces are commonly associated with nonpolar atom–atom interactions and hydrophobic protein interaction regions, whereas the Coulombic or electrostatic partial charge and charge interactions are commonly associated with hydrophilic protein interaction regions and can include hydrogen bonding and backbone atom partial charge interactions. Here we define hydrogen bonding as dominant electrostatic energetic interactions between H-X atoms at a separation distance of less than 2.0 angstroms.

### 2.4. Structure and Dynamics of Omicron’s Flap

In general, the stability of the isolated SARS-CoV-2 RBD associated with its ½ β barrel motif creates an isolated stable structure that, for example, has led to its use as a highly effective COVID-19 vaccine [29]. Here the flap dynamics of an isolated RBD are studied beginning with the RBD, Up-Chain conformation (residues C336-L518) of the trimeric spike protein parsed from published experimental structure files in a “One-Up” conformation [PDB ID: 7THK [20] (Omicron, BA.1), 7WK3 [30] (Omicron, BA.1), 6Z97 [22] (WT), 6VSB [21] (WT)]. This restriction of studying the isolated Up-Chain RBD allows for a more focused study on the dynamic behavior of its β hairpin loop domain and also the correlation of the RBD binding to ACE2 using experimental structure files that often consider the RBD in isolation from its parent spike protein. These experimental-based starting structures are then studied over a relatively long 0.3 μs time period (150 million time steps) abstracting static and dynamic information to ascertain key differences between WT and Omicron RBD. For the sake of completeness, we note that the “Down State” RBD and its interactions with its remaining S1 and S2 domains as well as neighboring chains have been studied previously [27] and are not considered here.

## 3. Results 

**Figure 2:**
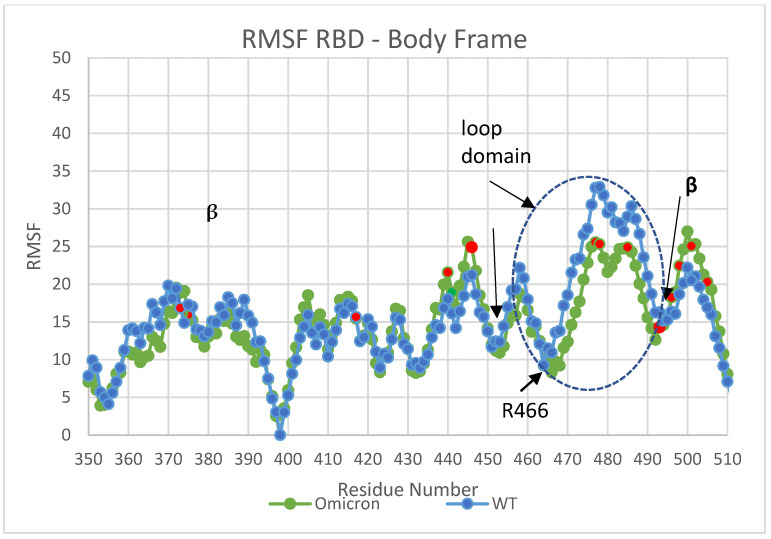
RMSF (C_α_ positions relative to the center of mass) values for Omicron (7THK [20]) versus WT (6Z97 [22]) showing a somewhat greater overall translational (internal) flexibility of WT versus Omicron (~10 to 15%). Mutated residues are shown in red.

Figure 2 compares the RMSF values for Omicron and WT after a 0.3 microsecond simulation. In general, the flap domain contributes the most to the molecular motion with an enhanced relative translational stability of the Omicron flap domain over WT.

Similar RMSF results demonstrating the greater flexibility of the β hairpin loop domain were obtained using PDB IDs: 6VSB [21] and 7WK3 [19], as shown in Figure S1 (Supplement S3). 

**Figure 3:**
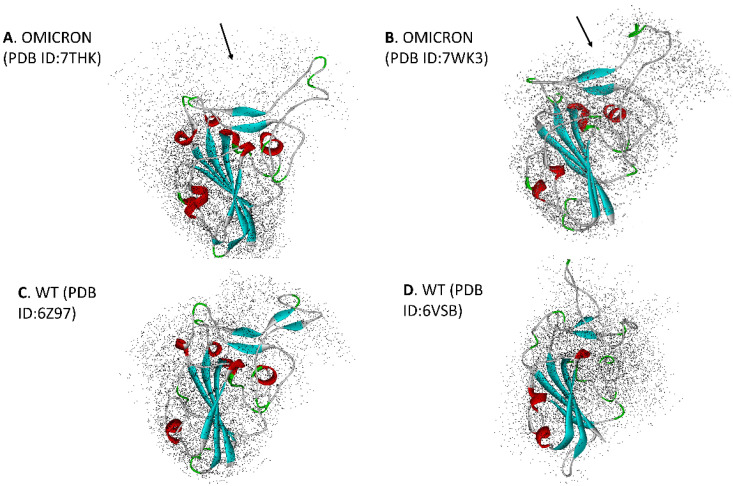
C-alpha positions shown over the last 50 nanoseconds (ns) of a 0.3 microsecond (μs) MD simulation. The more diffuse pattern of WT is evident. The average overall structure of the RDB over this period is shown in ribbons (Red: helices, blue: beta strands). (**A**) PDB ID: Omicron 7THK [20], (**B**) PDB ID: Omicron 7WK3 [30], (**C**) PDB ID: WT 6Z97 [22], (**D**) PDB ID: WT 6VSB [21].

Figure 3 shows the corresponding C-alpha positions from Figure 2 across the entire RBD over the last 50 ns. of MD simulation for both WT and Omicron RBD. The more diffuse vector positions in the flap domain are clearly shown for WT compared to Omicron. Note that the open flap conformation of Omicron is maintained throughout the entire 0.3 μs. simulation (Supplement S1). Although 7THK shows slightly more diffuse behavior than 7WK3, it is still more stable than WT. The complete computer visualization simulations over the entire time period are given in the Supplementary Material.

**Figure 4:**
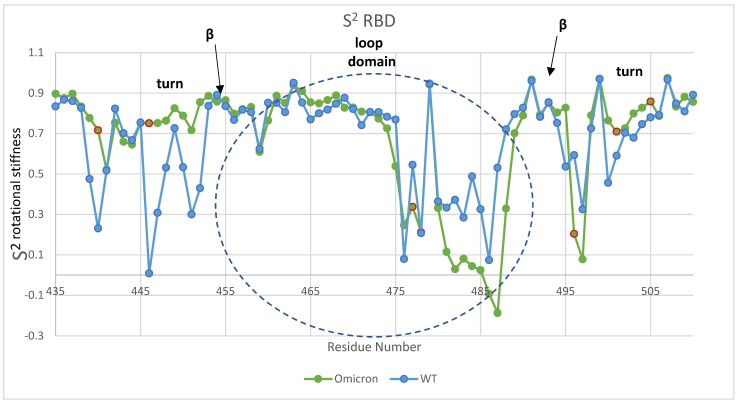
Order parameter, S^2^, for Omicron and WT in the loop domain. Values of S^2^ near 1 are indicative of rotational “stiffness” and those near zero are rotationally disordered states. The turn domains with mutations 441, 447, 501, and 505 demonstrate enhanced rotational stability for Omicron over WT. The negative order parameter shown for Omicron in the loop domain residues 486 and 487 is indicative of oscillations between states and not disorder. Note that one of the three disulfide bonds is between cysteine residues 480 and 488 for both WT and Omicron. The red circles are mutated residues.

Note that the differences between Omicron and WT shown in Figure 3 and the supplemental visualizations are also due to the rotational or orientational stability of the β hairpin loop domain. To further illustrate the orientational stability, the order parameter, which is the plateau value of the orientational correlation function for the backbone residue N-H bond vectors, is shown in Figure 4. The order parameter is similar in both cases with the important exception of the turn domains with mutations N440K and N501Y that demonstrate an enhanced rotational stability for Omicron over WT.

### Intra-Loop and Loop–RBD Interactions

**Figure 5:**
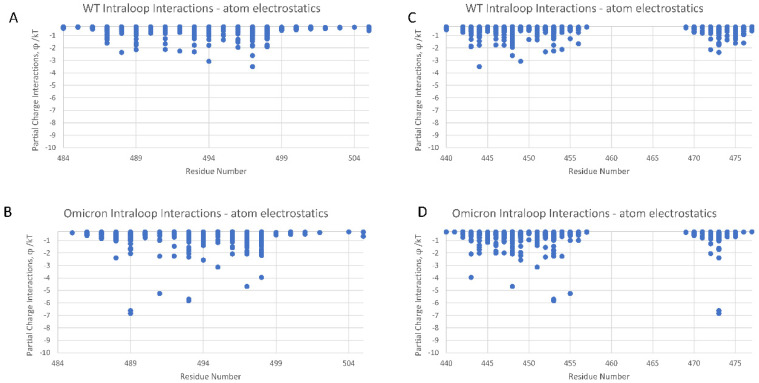
(**A**–**D**) Intra-loop residues with dominant energetic interactions showing the significant atom–atom interaction energy increase (φ/kT) for Omicron (PDB ID: 7THK) over WT (PDB ID: 6Z97).

**Figure 6:**
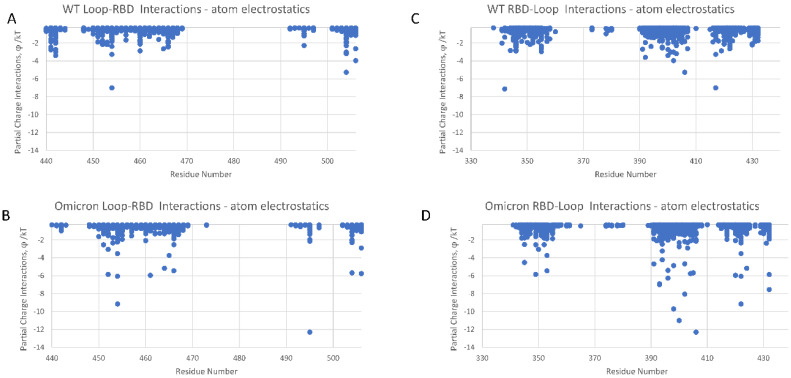
(**A**–**D**) Loop–RBD residues with dominant energetic interactions showing the significantly increased atom–atom interaction energies of Omicron (PDB ID: 7THK) over WT (PDB ID: 6Z97).

We conducted dominant energetic mappings, as described earlier, in order to pinpoint the precise new residue interactions that lead to the more stable internal translational and orientational state of the Omicron RBD loop domain (Figure 5 and Figure 6). As with WT, electrostatic interactions are the dominant type of interaction within the RBD. The stability of Omicron’s RBD is shown to be associated with increased interactions among the residues of the loop domain (intra-loop interactions: residues N439-S477 mapped to residues G485-H505) and loop–RBD interactions (residues C336-C432 mapped to residues N439-L518) (Figure 5 and Figure 6, respectively). The intra-loop interactions include three new hydrogen bonds between Y475 and E485, L455 and P491, and Y453 and R493 (Supplemental S2). The loop–RBD interactions include new hydrogen bonds between residues N422-R454, D420-L461, L424-F464, E406-Y495, and a significantly enhanced loop domain residue R466 interaction with residues A352-N354. This latter interaction is highly conspicuous in the RMSF data, as specifically marked in Figure 2, and plays a key role in maintaining the flap open state of Omicron’s RBD.

It is interesting that none of the ten RBD primary binding site mutations of Omicron are directly associated with the noncovalent atom–atom energy interaction increases shown in Figure 5 and Figure 6. So, mutations in the β hairpin loop domain translate into the stability of non-mutated residues.

**Figure 7:**
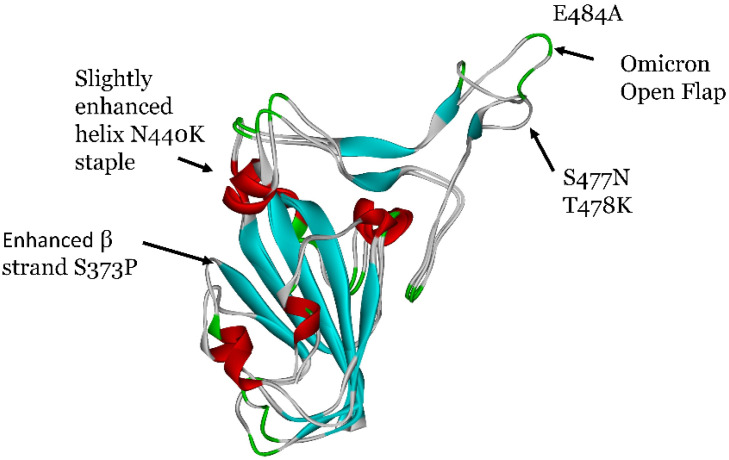
Rigid superimposition or alignment of the average structure of Omicron and WT RBD showing secondary structural motifs (Red: α helix, blue: β strand) for the last 50 ns of a 0.3 μs simulation.

To pinpoint the overall conformational changes, the average structures of Omicron and WT after 0.3 microseconds of total simulation were superimposed as shown in Figure 7.

The key conformational differences are the enhanced beta strands in the ½ beta-barrel structure due to S373P mutation and a slightly enhanced helix (one more helical residue) whereby the N440K creates a new so-called NK helical “staple” N437-K440. Note that the mutation K417N was not demonstrated to increase or decrease the helicity of its associated WT structure. As shown previously [13], the enhanced β strand due to S373P increases the stability of the ½ β-barrel structural motif and the overall mechanical stability of the Omicron RBD consistent with our observations here.

**Figure 8:**
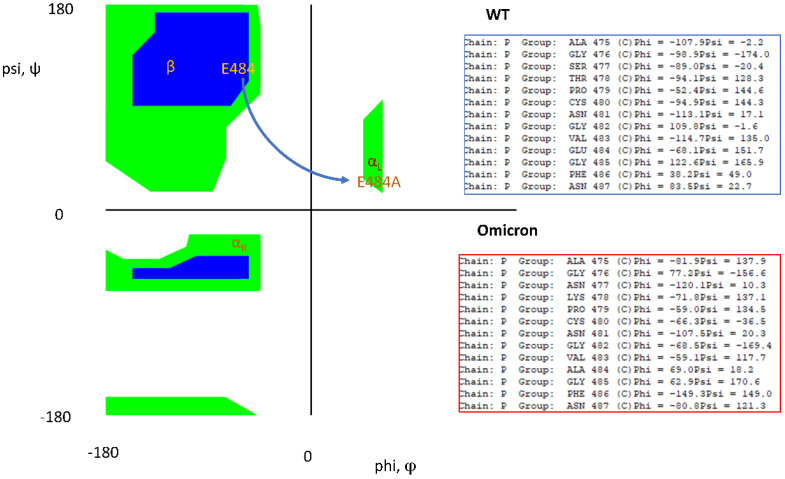
Key Ramachandran angle changes in the loop domain of WT versus Omicron. “Allowed” Ramachandran angles are shown in blue/green shaded areas. E484A shifts from its WT β sheet type orientation to a left-handed alpha helix orientation.

In addition to the importance of S373P, mutation E484A may help enhance the open flap conformation along with its enhanced ACE2 binding (Table 1). Specifically, the mutation E484A involves a significant Ramachandran angle shift from a β sheet domain to an atypical left-handed alpha helix domain as shown in Figure 8. Left-handed helices, although unusual, have been linked to stability and ligand binding [31]. It is observed that F486 and N487, which immediately neighbor the disulfide bond C488-C480, transition out of their α_L_ state from WT to Omicron, but are not involved in ACE2 binding (Table 1). 

**Figure 9:**
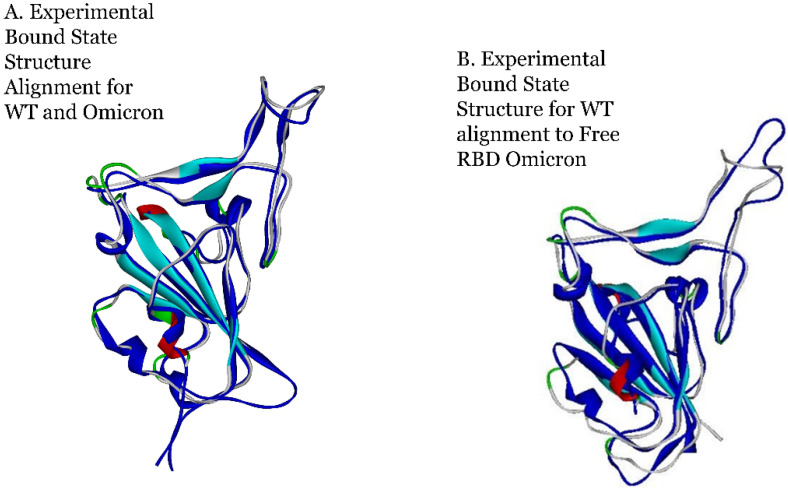
Superimposition of Omicron (navy) and WT (gray, aqua, blue, red). (**A**) Alignment of experimental structure files WT: 6M17 [23] bound RBD to ACE2 (not shown for clarity) and Omicron 7T9L [25] bound RBD to ACE2. (**B**) Alignment after 0.3 μs simulation WT: 6M17 [23] RBD bound to ACE2; Omicron 7THK unbound to ACE2 (“free”).

We also examined the ACE2 bound state to both WT (PDB ID: 6M17 [23]) and Omicron (PDB ID: 7T9L [25]) to investigate conformational differences in the bound states of WT and Omicron. As shown in Figure 9A, the experimental *bound state conformations* of the RBD are nearly identical between WT and Omicron. The ACE2 protein was then parsed from the Omicron experimental structure file and this unbound state of Omicron was allowed to re-equilibrate to its “free” state after 0.3 microseconds of simulation. Figure 9B shows the alignment of the dynamically obtained structure of Omicron with the bound state experimental structure of WT and demonstrates Omicron’s conformational likeness in its free state to the bound state conformation with ACE2. This was not observed previously for WT RBD with ACE2 due to the high mobility of the β hairpin loop structure [1]. 

Note that the alignment observed for the bound state structures of WT and Omicron was further verified with independent experimental structure files WT PDB ID: 6M0J [24] and Omicron PDB ID: 7U0N [26], as shown in Figure S1. 

## 4. Discussion

The β hairpin loop of the receptor binding domain of SARS-CoV-2 spike protein plays a critical role in its binding to ACE2, which initiates viral cellular entry. Through all-atom molecular dynamic simulations, we have previously shown that the flexibility in the loop domain can lead to the periodic obstruction of the primary ACE2 binding site [1]. In this study, it has been shown that mutations in this region associated with Omicron variants significantly increase the conformational stability of this complex loop structure. These increased interactions, which are primarily electrostatic due to partial charge interactions of residue atoms, include intra-loop interactions and interactions of the loop domain with the remaining core of the RBD. The intra-loop interactions include three new hydrogen bonds between Y475 and E485, L455 and P491, and Y453 and R493, and the loop–RBD interactions include new hydrogen bonds between residues N422-R454, D420-L461, L424-F464, and E406-Y495. In addition, there is a significantly enhanced loop domain residue R466 interaction with residues A352-N354. None of the residues associated with the increased interaction energies are Omicron mutated residues. Thus, the mutations translate into new, stabilizing conformations of non-mutated residues. Our results are in agreement with previous experimental studies demonstrating the enhanced stability of the core β barrel domain of the Omicron variant over its so-called wild-type (WT), or the initially sequenced virus of the SARS-CoV-2 outbreak [13]. There are a number of interesting features of the Omicron variant β hairpin loop domain, such as E484A, whereby the specific residues associated with Omicron’s mutations are on average not directly involved in its binding to ACE2, but rather help stabilize the loop conformation to a more stable or rigid open state. We have also shown that the average dynamic conformation of the unbound state of Omicron’s RBD is closely aligned to its bound state conformation to ACE2, which was not observed for WT. Concerning the recent sub-lineages of Omicron, such as XBB and JN.1, it is not expected that these limited additional mutations would significantly change the conformational stability results given here for the parent Omicron lineage.

This study raises questions on the possible “fitness” evolution of the Omicron variant, in addition to increased binding energies, to a more biomechanically stable conformation with a likeness to its bound state structure. As noted here, there are many factors associated with viral infection and transmission, and it is speculative to attribute the observed increases in the Omicron variant transmission to any one particular factor [14]. However, biomechanical stability could play a role through various pathways. For example, stability could increase the spike proteins’ overall conformational stability to both in vivo and environmental conditions of temperature, pH, etc. The more bound-state-like, stable conformation could help reduce entropic barriers to binding. However, what is clear is the now persistent Omicron RBD variant and subvariants are distinctly and significantly more biomechanically stable than its initial SARS-CoV-2 outbreak form. More practically, these changes toward the biomechanical stability of the Omicron variant may be noteworthy, in general, as providing possible strategies for improving protein stability, for example, as improved, more stable antigens for vaccines through “engineered” mutations [21,32,33]. 

## Figures and Tables

**Table 1:** Omicron (BA.2) mutations in the *primary* ACE2 binding region as compared to the dominant energetic contact list.

ACE2-RBD Omicron Binding Region Mutations	Dominant Energetic Residue (Table S3 of Ref. [1])	Primary ACE2 Interacting Residues with WT Wuhan (Table S3 of Ref. [1])
N440K	Yes	Q30
G446S, BA.1 only	Yes	D38, Q42
S477N	Yes	D38, Q42
T478K	Yes	Q24
E484A	Yes	Q42, K31
Q493R	Yes	K31, E35
G496S, BA.1 only	Yes	D38, K351
Q498R	Yes	Y41
N501Y	Yes	G354
Y505H	Yes	K353

For BA.4 and BA.5 variants, two additional mutations (L452R and F486V) have ubiquitously appeared in the primary binding region. For the XBB Omicron subvariants, S486P is additionally present in the primary ACE2 binding region of these strains. For the recent JN.1 Omicron subvariants, L455S is an additional mutation in the receptor binding domain. Other mutations in the RBD (residues 336–518) include G339D, S371L (BA.1), S371F (BA.2), S373P, S375F, T376A (BA.2), D405Y(BA.2), and R408S (BA.2).

## Data Availability

All data are given in the Supplementary Material provided.

## Supplementary Materials

The following supporting information can be downloaded at https://www.mdpi.com/article/10.3390/v16060912/s1. Supplement S1 Movie. Movie files: 6Z97RBD.mp4 7THKRBD.mp4; Supplement S2 Mappings Energetic Mappings—Dominant atom–atom interactions data for Figure 7 and Figure 8. For formatting, please see supporting information in the OpenContact reference. 6Z97, Intra-loop interactions, 6Z97IntraLoopRBD-finedata.txt, Loop–RBD, 6Z97LoopRBD-finedata.txt, 7THK, Intra-loop interactions, 7THKIntraLoop-finedata.txt, Loop–RBD, 7THKLoopRBD-finedata.txt, 7UON, Supplement S3 Figure S1; Supplement S4 Tumbling Corrections. Tumbling Corrections.pdf; Supplement S5 Programming Details, rmsdnmrv2.f.
