# Supplementary material for: Mutations in the Receptor Binding Domain of Severe Acute Respiratory Coronavirus-2 Omicron Variant Spike Protein Significantly Stabilizes Its Conformation
Source: Viruses. 2024 Jun 4;16(6):912. doi: 10.3390/v16060912 (PMC11209484; doi:10.3390/v16060912)
Supplement: Supplementary file 1 [file viruses-16-00912-s001.zip › Figure S1.pdf]

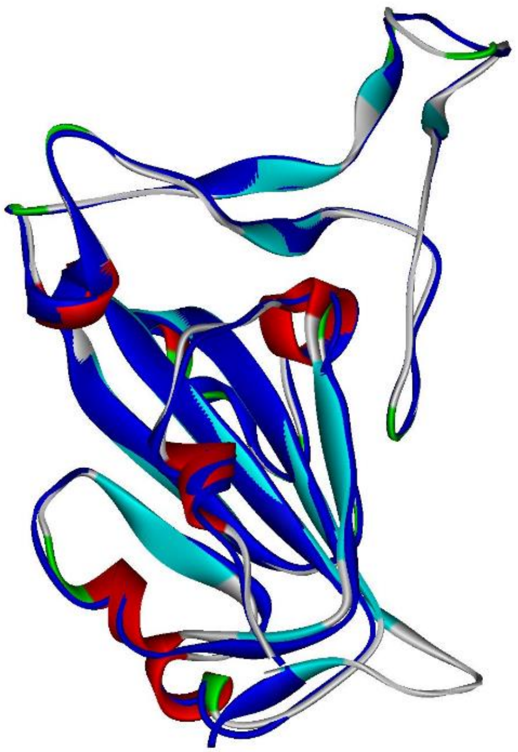

Figure S1. Alignment of RBD bound state structures to hACE2. Omicron (Navy, PDB ID: 7U0N[22]) and WT (Aqua Blue, Red, Gray, PDB ID: 6M0J[20]).
