# Supplementary material for: Mutations in the Receptor Binding Domain of Severe Acute Respiratory Coronavirus-2 Omicron Variant Spike Protein Significantly Stabilizes Its Conformation
Source: Viruses. 2024 Jun 4;16(6):912. doi: 10.3390/v16060912 (PMC11209484; doi:10.3390/v16060912)
Supplement: Supplementary file 1 [file viruses-16-00912-s001.zip › Tumbling Corrections.pdf]

S4. Tumbling Corrections

We can exploit the fact that the ½ beta barrel region is approximately a rigid body and examine internal rotations relative to this rigid body motion (tumbling or macromolecular rotational diffusion). In general, any rigid body motion can be divided between translation of the center of mass (COM) and rotation about the COM. Consider two different protein orientations relative to the COM as shown below:

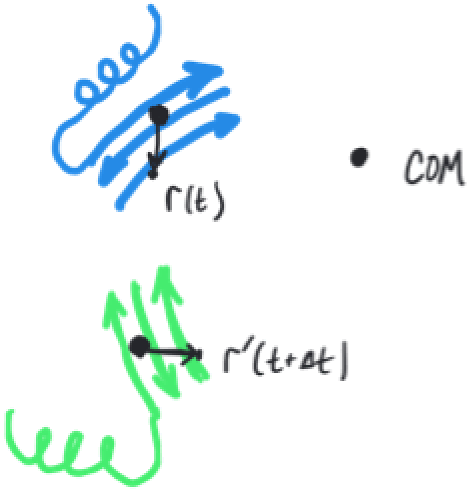

For orientations, the position vector for any point (atom) of the rigid body (protein) at time (t+Δt), **r**′(t+Δt), is related to its former orientation at time t, **r**(t), through the rotational transformation matrix **A** by

$$\mathbf{r}'\,(t+\Delta t) = \mathbf{A}\,\mathbf{r}\,(t) \qquad (1)$$

To correct for the tumbling rotation, the inverse process is needed, or from Eq. (1)

$$\mathbf{A}^{-1}\,\mathbf{r}' = \mathbf{r} \qquad (2)$$

We can determine the inverse transformation matrix $\mathbf{A}^{-1}$ as follows:

1. First take the transpose of Eq. (2) to reverse the operation order

$$\mathbf{r}^{'\mathbf{T}}\,\mathbf{B}^{T} = \mathbf{r}^{\mathbf{T}} \qquad (3)$$

where $\mathbf{B} = \mathbf{A}^{-1}$.

2. Now multiply through Eq. (3) by the inverse of $\mathbf{r}'^{T}$

$$\left(\mathbf{r}'^{\mathbf{T}}\right)^{-1}\mathbf{r}'^{T}\,\mathbf{B}^{T} = \mathbf{B}^{T}$$
$$= \left(\mathbf{r}'^{\mathbf{T}}\right)^{-1}\mathbf{r}^{\mathrm{T}} \qquad (4)$$

3. Finally,

$$\left(\mathbf{B}^{\mathbf{T}}\right)^{\mathbf{T}} = \mathbf{B} \qquad (5)$$

The unknown matrix $\mathbf{B}^{\mathbf{T}}$, which involves 9 elements, can be determined from Eq. (4) and knowledge of any three points in the rigid body at times t and t+Δt. Then all points (atoms) of the protein can be rotated using Eq. (2). These new positions are then used to determine the (internal) rotational correlation function with tumbling correction.

Programming details are given in Supplement 5 (S5).
